# Supplementary material for: Novel Driver Strength Index highlights important cancer genes in TCGA PanCanAtlas patients
Source: PeerJ. 2022 Aug 11;10:e13860. doi: 10.7717/peerj.13860 (PMC9375969; doi:10.7717/peerj.13860)
Supplement: Supplemental Information 2 [file peerj-10-13860-s002.zip › COHORTS/genes plots/age/2021_8_16_14_9_distribution_cohorts_females_85_.pdf]

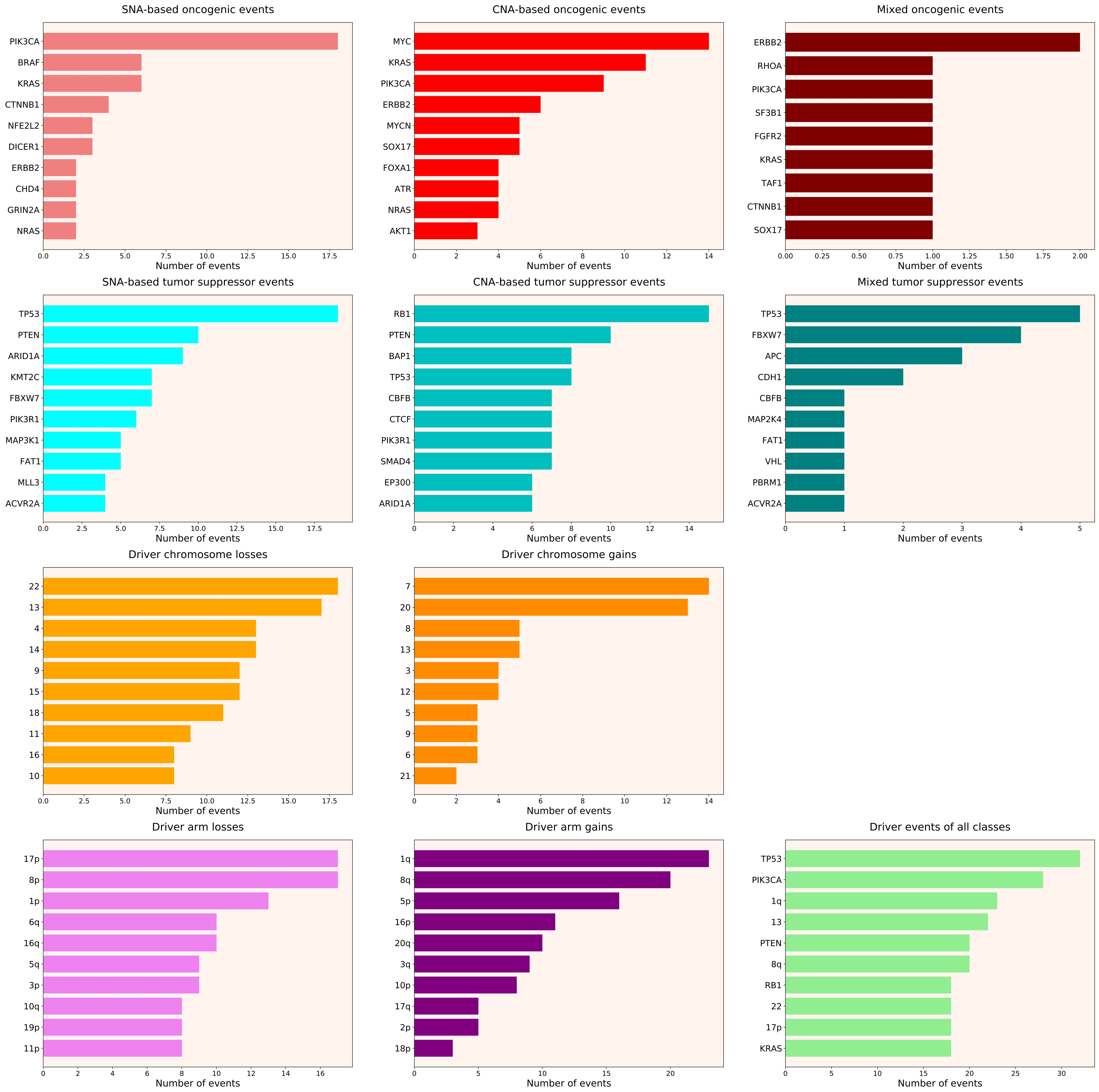
SNA-based oncogenic events
PIK3CA
BRAF
KRAS
CTNNB1
NFE2L2
DICER1
ERBB2
CHD4
GRIN2A
NRAS
Number of events
CNA-based oncogenic events
MYC
KRAS
PIK3CA
ERBB2
MYCN
SOX17
FOXA1
ATR
NRAS
AKT1
Number of events
Mixed oncogenic events
ERBB2
RHOA
PIK3CA
SF3B1
FGFR2
KRAS
TAF1
CTNNB1
SOX17
Number of events
SNA-based tumor suppressor events
TP53
PTEN
ARID1A
KMT2C
FBXW7
PIK3R1
MAP3K1
FAT1
MLL3
ACVR2A
Number of events
CNA-based tumor suppressor events
RB1
PTEN
BAP1
TP53
CBFB
CTCF
PIK3R1
SMAD4
EP300
ARID1A
Number of events
Mixed tumor suppressor events
TP53
FBXW7
APC
CDH1
CBFB
MAP2K4
FAT1
VHL
PBRM1
ACVR2A
Number of events
Driver chromosome losses
22
13
4
14
9
15
18
11
16
10
Number of events
Driver chromosome gains
7
20
8
13
3
12
5
9
6
21
Number of events
Driver arm losses
17p
8p
1p
6q
16q
5q
3p
10q
19p
11p
Number of events
Driver arm gains
1q
8q
5p
16p
20q
3q
10p
17q
2p
18p
Number of events
Driver events of all classes
TP53
PIK3CA
1q
13
PTEN
8q
RB1
22
17p
KRAS
Number of events
